# Letter to editor about “Association between socioeconomic status and multimorbidity indices across 15 countries: a multi-regional cohort study”

**DOI:** 10.1097/JS9.0000000000002833

**Published:** 2025-06-26

**Authors:** Hujun Wang, Yuling Wang, Congxiao Wang

**Affiliations:** a Department of Rehabilitation, Beijing Rehabilitation Hospital, Capital Medical University, Beijing, China; b School of Health Policy and Management, Chinese Academy of Medical Sciences & Peking Union Medical College, Beijing, China; c Department of the Second Orthopaedics Rehabilitation Centre, Beijing Rehabilitation Hospital, Capital Medical University, Beijing, China

*Dear Editor,*

I have read with great interest the recent article published in the International Journal of Surgery titled *“Association between socioeconomic status and multimorbidity indices across 15 countries: a multi-regional cohort study”*^[1]^. This study, drawing on data from four major international longitudinal cohorts (HRS, ELSA, SHARE, and CHARLS), explores the relationship between socioeconomic status (SES) and multimorbidity, offering valuable empirical evidence on global health inequalities. While acknowledging the importance of this work, I would like to offer several methodological and interpretative considerations for further discussion.

## Simplified SES classification may overlook national and cultural contexts

The authors dichotomized SES into “high” and “low” categories to harmonize data across cohorts. While this approach facilitates cross-national comparison, it may obscure important structural and cultural differences between countries. For instance, China’s urban–rural divide, retirement system, and labor market dynamics may alter how education, income, and employment relate to health compared to Western contexts. A simple binary classification might not adequately capture these complexities. Future analyses could benefit from adopting country-specific SES measures or multilevel models incorporating national-level interactions to enhance explanatory power and international comparability.

## Variability in multimorbidity measures and lack of mental/cognitive disorder stratification

The use of five different multimorbidity indices (Charlson, Elixhauser, HRQoL, MWI, and simple counts) demonstrates methodological rigor, yet these indices vary considerably in the chronic conditions they include, particularly regarding mental and cognitive disorders. Given that individuals with lower SES are more vulnerable to depression, anxiety, and cognitive impairment^[2]^, insufficient adjustment for these conditions may underestimate the SES–morbidity association. Moreover, while the use of multiple indices enhances robustness, their original clinical purposes differ. For example, the Charlson Index is designed to predict short-term mortality, not to comprehensively assess chronic disease burden^[3]^.

## Lack of absolute risk measures limits policy relevance

Although the study reports hazard ratios for multimorbidity across SES groups, it does not present absolute rate differences or attributable risks – metrics that are crucial for policy decision-making. For example, a high HR may not translate into a meaningful population-level impact if the absolute incidence difference is small. Including these measures would allow for better prioritization of public health interventions and more informed resource allocation.

## Omission of health behaviors raises concern for residual confounding

The analysis does not adjust for key lifestyle factors such as smoking, alcohol use, physical activity, and diet, which are strongly associated with both SES and multimorbidity^[4]^. Although such data may be difficult to collect consistently across large cohorts, their absence raises the possibility of residual confounding. It would be valuable for the authors to acknowledge this limitation and encourage future studies to incorporate these potential mediators and moderators in analytic models.

In conclusion, this study makes an important contribution to understanding the social determinants of multimorbidity on a global scale. Nonetheless, refinements in SES classification, multimorbidity measurement, and risk communication would further strengthen the findings^[5]^. We commend the authors’ efforts and encourage future research to enhance SES modeling flexibility, provide nuanced classification of chronic diseases, and incorporate both relative and absolute risk metrics to better inform global health equity strategies.

## Data Availability

Not applicable.
